# Active site plasticity revealed from the structure of the enterobacterial N-ribohydrolase RihA bound to a competitive inhibitor

**DOI:** 10.1186/1472-6807-10-14

**Published:** 2010-06-08

**Authors:** Gianpiero Garau, Laura Muzzolini, Paola Tornaghi, Massimo Degano

**Affiliations:** 1 From the Biocrystallography Unit, Division of Immunology, Transplantation, and Infectious Diseases - Scientific Institute S. Raffaele, via Olgettina 58, 20132 Milan - Italy; 2 Drug Discovery and Development Unit - Italian Institute of Technology. Via Morego 30. 16163 Genoa - Italy

## Abstract

**Background:**

Pyrimidine-preferring N-ribohydrolases (CU-NHs) are a class of Ca^2+^-dependent enzymes that catalyze the hydrolytic cleavage of the N-glycosidic bond in pyrimidine nucleosides. With the exception of few selected organisms, their physiological relevance in prokaryotes and eukaryotes is yet under investigation.

**Results:**

Here, we report the first crystal structure of a CU-NH bound to a competitive inhibitor, the complex between the *Escherichia coli *enzyme RihA bound to 3, 4-diaminophenyl-iminoribitol (DAPIR) to a resolution of 2.1 Å. The ligand can bind at the active site in two distinct orientations, and the stabilization of two flexible active site regions is pivotal to establish the interactions required for substrate discrimination and catalysis.

**Conclusions:**

A comparison with the product-bound RihA structure allows a rationalization of the structural rearrangements required for an enzymatic catalytic cycle, highlighting a substrate-assisted cooperative motion, and suggesting a yet overlooked role of the conserved His82 residue in modulating product release. Differences in the structural features of the active sites in the two homologous CU-NHs RihA and RihB from *E. coli *provide a rationale for their fine differences in substrate specificity. These new findings hint at a possible role of CU-NHs in the breakdown of modified nucleosides derived from RNA molecules.

## Background

Pyrimidine-preferring nucleoside hydrolases (CU-NHs) are members of the broad family of Ca^2+^-dependent hydrolases that catalyze the cleavage of the N-glycosidic bond in nucleosides [1,2]. Enzymes with NH activity have been isolated from several different organisms, ranging from bacteria to fungi, nematodes, insects, and plants [3-7]. The biological function of these enzymes in both prokaryotes and eukaryotes is still controversial, and the widespread presence of NH-encoding genes can either be ascribed to a conserved role in general nucleotide catabolism. For instance, several purine or pyrimidine-auxotrophic parasites such as protozoa rely on nucleoside hydrolases to recycle nitrogenous bases uptaken from the host, lacking nucleoside phosphorylase activity [7]. In addition, NHs are also apparently involved in species-specific processes. Purine-specific NHs appear to modulate sporulation in spore-forming bacteria, such as *Bacillus cereus *and *Bacillus anthracis *[5], or to promote host anaesthesia during micropredation by blood-sucking insects such as *Aedes aegypti *[8]. In the yeast *Saccharomyces cerevisiae*, the URH1 uridine hydrolase displays a highly selective pyridine nucleosidase activity towards nicotinamide riboside that is instrumental for NRK1-independent NAD^+ ^synthesis [9,10].

In *Escherichia coli *nucleoside phosphorylases catalyze the scission of the N-glycosidic bond in the most common RNA nucleosides. The presence of two enzymes with CU-NH activity has been proposed to provide considerable flexibility for mRNA degradation in different environmental conditions, or the ability to hydrolyze some low-level modified nucleoside of tRNA and rRNA [3,11]. Indeed, the enterobacterial RihB CU-NH is active on synthetic 5-substituted uridines, and thus may also act on similarly modified nucleosides found in RNA molecules. More than one hundred naturally-occurring modifications have been so far observed in RNA nucleosides, playing important roles in the structural stability and function of RNA in all kingdoms of life [12,13]. However, RNA modifications can be introduced also by exogenous alkylating agents [14] or associated to antibiotic sensitivity [15], which alter the integrity and function of cellular RNAs (i.e. ribosome trapping and formation of truncated proteins). Despite the fact that nucleotide modification processes and effects have been the subject of intense research during last years, the catabolic pathways of modified nucleosides still requires investigation. A tempting possibility is that CU-NHs may provide a primary degradation pathway for such modified nucleic acids components. Hence, further insights into the substrate specificity of bacterial CU-NH, together with the definition of the structural determinants involved in substrate binding, are required to validate this hypothesis.

CU-NHs are homologous to the non-specific IU-NH isozymes at central active site positions, and thus can be classified as belonging to a common homology group, termed NH Group I [11]. A recent mutagenesis study demonstrated that the absence of two specific active site tyrosines in CU-NHs is responsible for the slow turnover of purine nucleosides [16]. Two CU-NH-encoding genes are present in *E. coli *cells, termed either *ybeK *and *yeiK*, or *rihA *and *rihB*, respectively. The *rihB *gene was shown as physiologically silent, but its transcription is raised 25-fold in autoinducer-2 conditioned medium [17]. The *rihA *gene is poorly expressed only in glucose-rich medium [3,17]. The gene products RihA and RihB have been characterized using steady-state kinetics and X-ray crystallography, and display similar kinetic constants, characterized by mid- to high-micromolar K_M _values for uridine and cytidine [16,18,19]. RihB can also hydrolyze the N-glycosidic bond in 5-substituted uridines, reinforcing the hypothesis of a broad substrate specificity to include modified RNA nucleosides. The crystal structure of RihB has been determined both in presence of the fortuitous ligand glycerol [11], and of the slowly-hydrolyzed purinic substrate inosine [16], while RihA was co-crystallized with the reaction product ribose [19]. These structures remarked the overall similarity with IU-NHs in both the protein fold and ribosyl binding site. The ribose-bound RihA displays an open structure suggesting that a ligand mimicking the nitrogenous base is required for enzyme closure. In particular, two active site loops connecting strand β3 to helix α3 (loop αL) and the C-terminal portion of helix α9 were highly flexible and not traceable in electron density maps. A single histidine residue (His13) was observed in two discrete orientations, and the transition between these two conformations was postulated as crucial in allowing the rearrangement between the open and closed forms. Yet, the nature of the interactions stabilizing the closed form structure, and the events following chemistry that lead to the active site opening remain largely unknown.

Here, we report the crystal structure of the complex between the *E. coli *RihA enzyme and the competitive inhibitor 3,4-diamino phenyliminoribitol (DAPIR) to a resolution of 2.1 Å. The compound resembles the geometric and electronic features of the transition state of the reaction catalyzed by the homologous IU-NH from *C. fasciculata*. Binding of the ligand to the active site of RihA results in the rearrangement of the αL and α9 segments through an induced fit mechanism that allows the interaction of the enzyme with the nitrogenous base-mimicking moiety of the ligand. Interestingly, the residues involved in interactions with the nitrogenous base differ between the highly homologous CU-NHs RihA and RihB from *E. coli*, suggesting that comparable levels of transition state stabilization for the hydrolysis of pyrimidine nucleosides can be achieved through qualitatively different interactions. A comparison with the structure of the reaction product-bound RihA provides snapshots of the enzyme structure during pyrimidine nucleoside hydrolysis, and identifies the central interactions that drive the transition from the open to the closed conformation. The structure of the complex underscores the adaptability of the NH active site, showing how the active site of RihA can adapt to different ligands and accommodate several modified nitrogenous bases, and thus supporting the hypothesis of a role of CU-NHs extending to the catabolism of modified RNA nucleosides.

## Results

### Structure of the RihA-DAPIR complex

**Figure 1:**
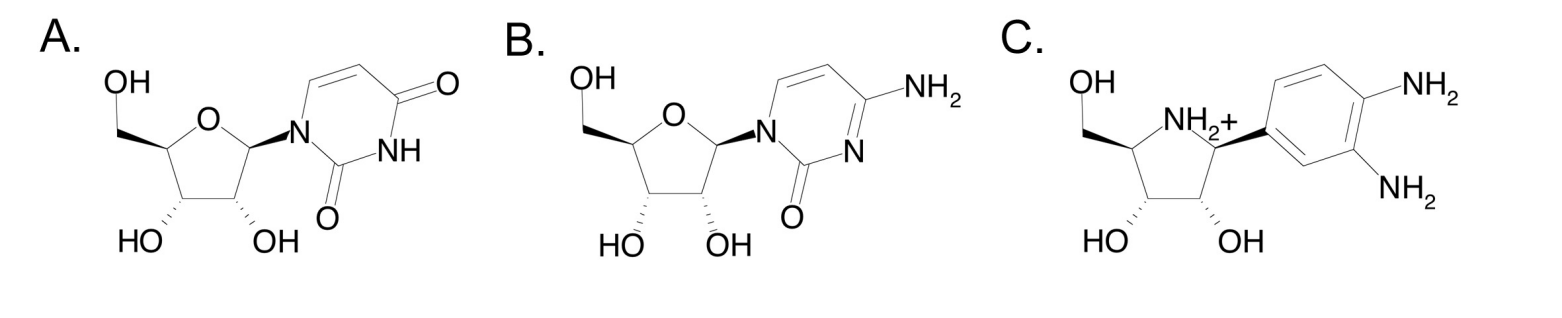
**Ligands of the *E. coli *pyrimidine-preferring NH RihA**. A) Uridine and B) cytidine, the natural substrates. C) The competitive inhibitor DAPIR. The iminoribitol moiety resembles the features of the transition state of the hydrolytic reaction, including a partial double bond character of the C1'-O4' bond, an elongated N-glycosidic bond, and a partial positive charge at the sugar ring.

The requirement of a detailed description of the NH-substrate contacts for the design of antiprotozoan compounds has prompted both experimental and theoretical studies [16,20-26]. The purpose of this work was the characterization a CU-NH enzyme bound to a competitive inhibitor in order to gain further insights into the specificity of pyrimidine-preferring NHs. The RihA enzyme is a good candidate for such structural analysis since it has been characterized in complex with the product of the hydrolytic reaction [19], and can thus yield detailed information on the relevant steps of the CU-NH catalytic cycle. We identified the compound 3,4-diamino phenyliminoribitol (DAPIR, Fig. 1) as a competitive inhibitor of RihA, with a K_I _= 85 ± 7 μM. RihA was co-crystallized bound to DAPIR, and the structure of the complex was determined at a resolution of 2.1 Å (Table 1). 1'-substituted iminoribitols are tight-binding, competitive inhibitors of several NH enzymes. The DAPIR molecule structurally resembles pyrimidine nucleosides, albeit lacking the O2 carbonyl that has been postulated as essential for catalysis [19]. Imiboribitols also mimic the transition state structure of the reaction catalyzed by the IU-NH enzyme from *C. fasciculata *[27]. The molecular replacement method unambiguously identified four independent molecules in the asymmetric unit, arranged as a tetramer with the subunits related by 222 symmetry. The final model comprises the four RihA polypeptides, and due to flexibility residues 225 through 229 in chain A, 76 to 85 and 225 to 230 in chain B, 76 to 85 and 223 to 229 in chain C, and 77 to 84 in chain D were omitted. These amino acids belong to the αL loop and α9 helix, that were disordered in the RihA-ribose complex. Residues Gly38 in all chains (as in other NH enzymes), Gly89 and Phe223 of chain B (both highly flexible) are outside the 99.8% regions of the Ramachandran plot.

**Table 1:** Crystallographic data

*Data collection*		
Resolution range (Å)	37.5-2.1	2.21-2.1

Number of observed reflections	232,754	31,773

Number of unique reflections	68,457	9,929

Completeness (%)	97.2	95.4

Multiplicity	3.4	3.2

Mean I/σ(I)	8.1	3.0

R_sym_	0.122	0.388

Refinement		

Resolution range (Å)	37.50-2.1	2.15-2.1

Number of reflections (F > 0)	65,238	4,760

R_crys_	0.203	0.254

R_free_	0.241	0.304

rmsd bonds (Å)	0.020	

rmsd angles (°)	1.689	

Mean B factor (Å^2^)	30.3	

Model quality		

Residues in favored regions of Ramachandran plot (%)	97.8	

Residues outliers in Ramachandran plot (%)	0.5	

Unusual rotamers (%)	0.9	

Data collection and refinement statistics for the RihA-DAPIR complex.

R_sym _= ∑_*hkl*_∑_i_||I_i_(*hkl*)|-|<I(*hkl*)||>|/∑_hkl_∑_i_|I_i_(*hkl*)|, where I_i_(*hkl*) is the ith measurement of reflection *hkl*, and <I(*hkl*)> is the average value from the individual measurements

R_crys _= ∑*_hkl_*||F_o_(*hkl*)|-|F_c_(*hkl*)||/∑*_hkl_*|F_o_(*hkl*)|, where F_o_(*hkl*) and F_c_(*hkl*) are the measured and calculated structure factor of reflection hkl, respectively

R_free_, same as R_crys_, but calculated on a subset of randomly-selected reflection excluded from all stages refinement (3,455 total, 265 in the highest resolution shell).

**Figure 2:**
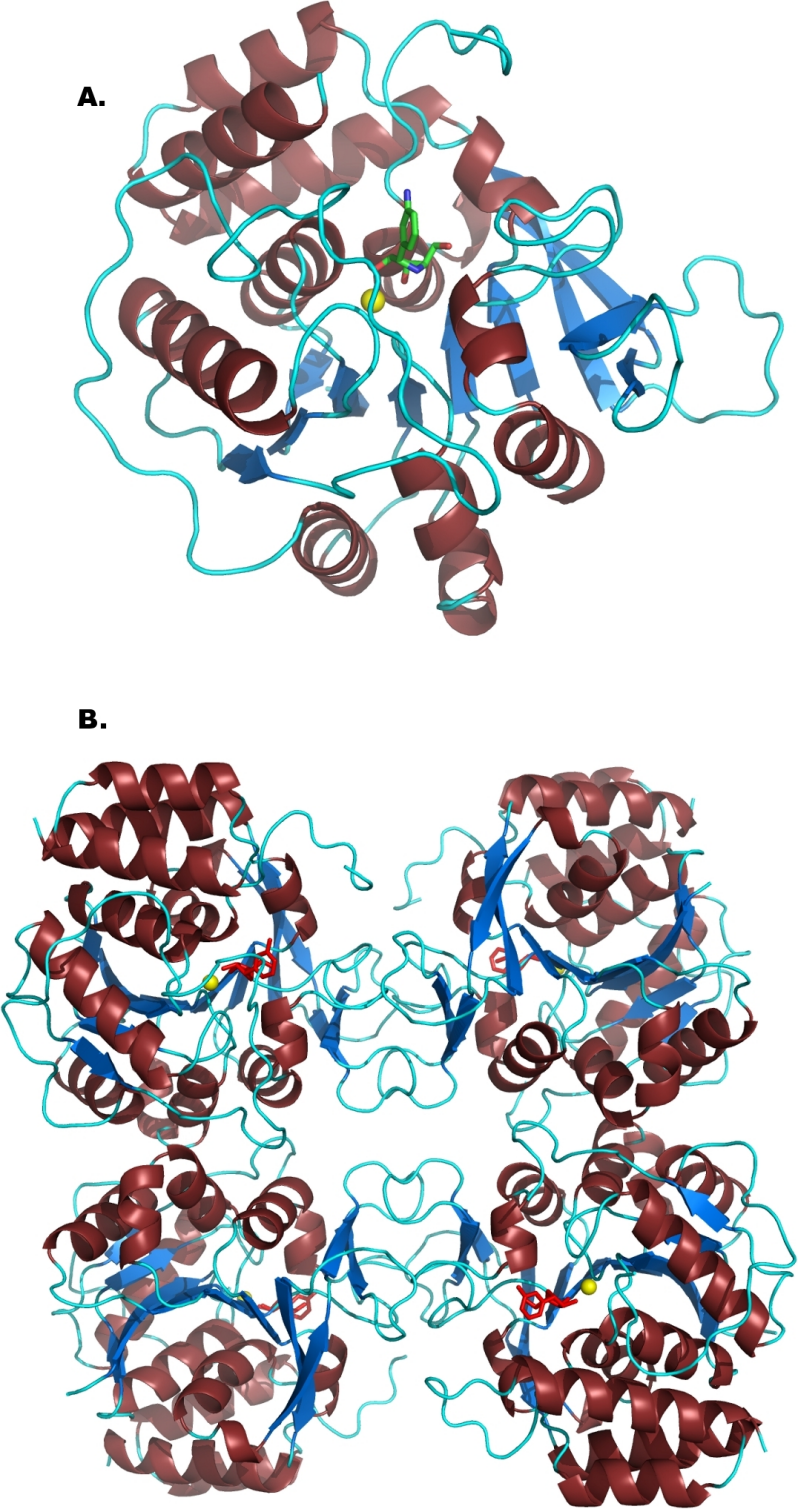
**Crystal structure of RihA bound to DAPIR**. A) The RihA monomer, with α-helices coloured brown, β-strands blue, and loop regions in cyan. The active site Ca^2+ ^ion is shown as a yellow sphere, and the DAPIR molecule as sticks coloured according to atom type. B) The RihA tetramer, as observed in the asymmetric unit of the RihA-DAPIR complex crystals.

The structure of each monomer maintains the α/β fold that is typical of NHs, with the Ca^2+^-containing active site located in a deep, narrow cavity at the C-terminal end of a core 10-stranded β-sheet surrounded by α-helices (Fig. 2A). The functional tetramer is identical within experimental error to that observed in the RihA-ribose orthorhombic crystals (rmsd = 0.61 Å considering 1,208 Cα atoms), thus showing that the binding of different ligands does not induce significant changes in the quaternary structure (Fig. 2B). Furthermore, the interface area between two dimer subunits remains unaltered when comparing the ribose-bound and the DAPIR-bound complexes. The only relevant difference occurring at the dimer interface involves the C-terminal portion of helix α9 of one subunit and the turn connecting the strands β11 and β12 of the proximal one. The side chain of Trp232 rotates slightly around its side chain torsion angle χ^1 ^(from -52° to -76°) to establish a π-π stacking interaction with the aromatic ring of Tyr279 from the second subunit. Moreover, Lys231 is at H-bond distance with the carbonyl groups of Tyr279 and Tyr280. These interactions are present in each subunit, and therefore provide significant stabilization to the RihA quaternary structure when a ligand is bound at the active site.

### Binding of a competitive inhibitor to the RihA CU-NH

**Figure 3:**
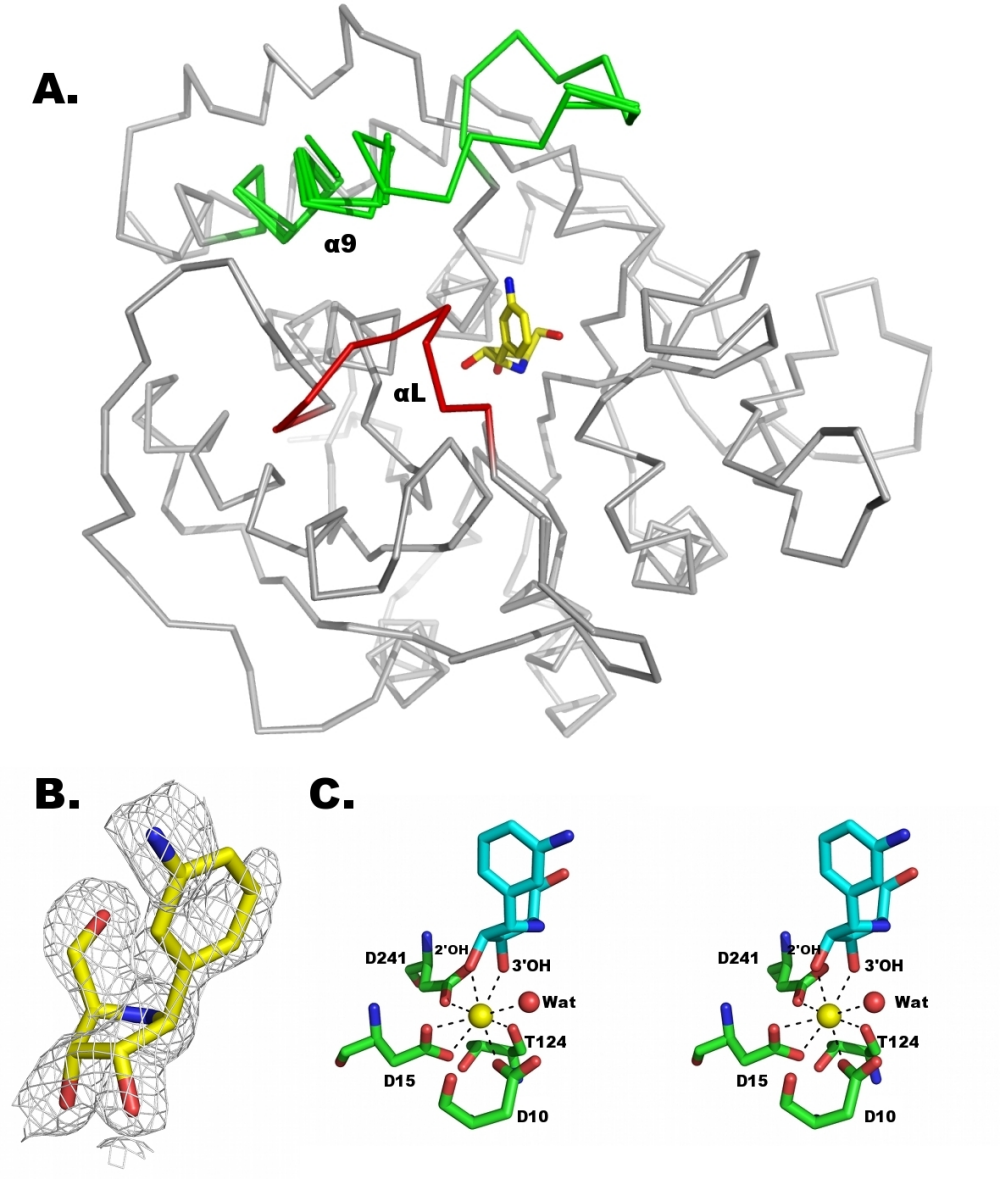
**Structural features of the RihA-DAPIR complex**. A) Superposition of the four crystallographically-independent molecules of RihA. The Cα trace of the four RihA monomers are shown in grey, and the αL (red) and α9 (green) segments are labelled for clarity. The four independent RihA chains are virtually undistinguishable. The αL loop is completely visible in chain A, while the full α9 helix could be traced only in chain D, as per nomenclature of the deposited coordinates. B) The DAPIR molecule bound to the active site of chain A superimposed with a (2mF_o_-DF_c_, ϕ_c_) shake-omit electron density map contoured at 1.2σ. C) Octacoordination of the active site Ca^2+ ^ion. Five oxygen atoms from RihA amino acid residues, the O2' and O3' hydroxyls of DAPIR, and an highly-ordered water molecule complete the coordination sphere of the metal.

The electron density map showed the presence of one inhibitor molecule in each of the monomers in the crystal asymmetric unit. The αL loop of RihA undergoes a tightening around the inhibitor only in chain A, while the α9 helix is completely traceable in electron density maps in chain D (Fig. 3A and 3B). The DAPIR molecule is bound at the RihA active site in two conformations, differing in the orientation of the diaminophenyl group. The N3 amino group of DAPIR can either be directed towards the α9 helix of RihA (as found in chain A, as per PDB file nomenclature), or towards the αL loop (chains B and D). These two conformations are related by a 180° rotation about the C-C pseudo-glycosidic bond. In chain C, the inhibitor is bound in both conformations, with approximately equal occupancy.

In agreement with the mode of binding of ribonucleoside analogues to group I NHs observed by X-ray crystallography [16,24], the binding between DAPIR and the calcium metal centre in RihA occurs through the 2' and 3'-hydroxyl groups of the ribose moiety, and is further strengthened by hydrogen bonding of the hydroxyl group in O5' to Asn158 and Glu164. The octacoordination of the calcium ion involves the DAPIR 2' and 3'-hydroxyls, the carboxylates of three Asp residues at positions 10, 15, and 241, the Thr124 backbone oxygen, and a water molecule (Fig. 3C). This ordered water molecule is hydrogen bonded to both Asp10 and Asn166, and reveals the position of the incoming nucleophile in the CU-NH-catalyzed hydrolytic reaction.

**Figure 4:**
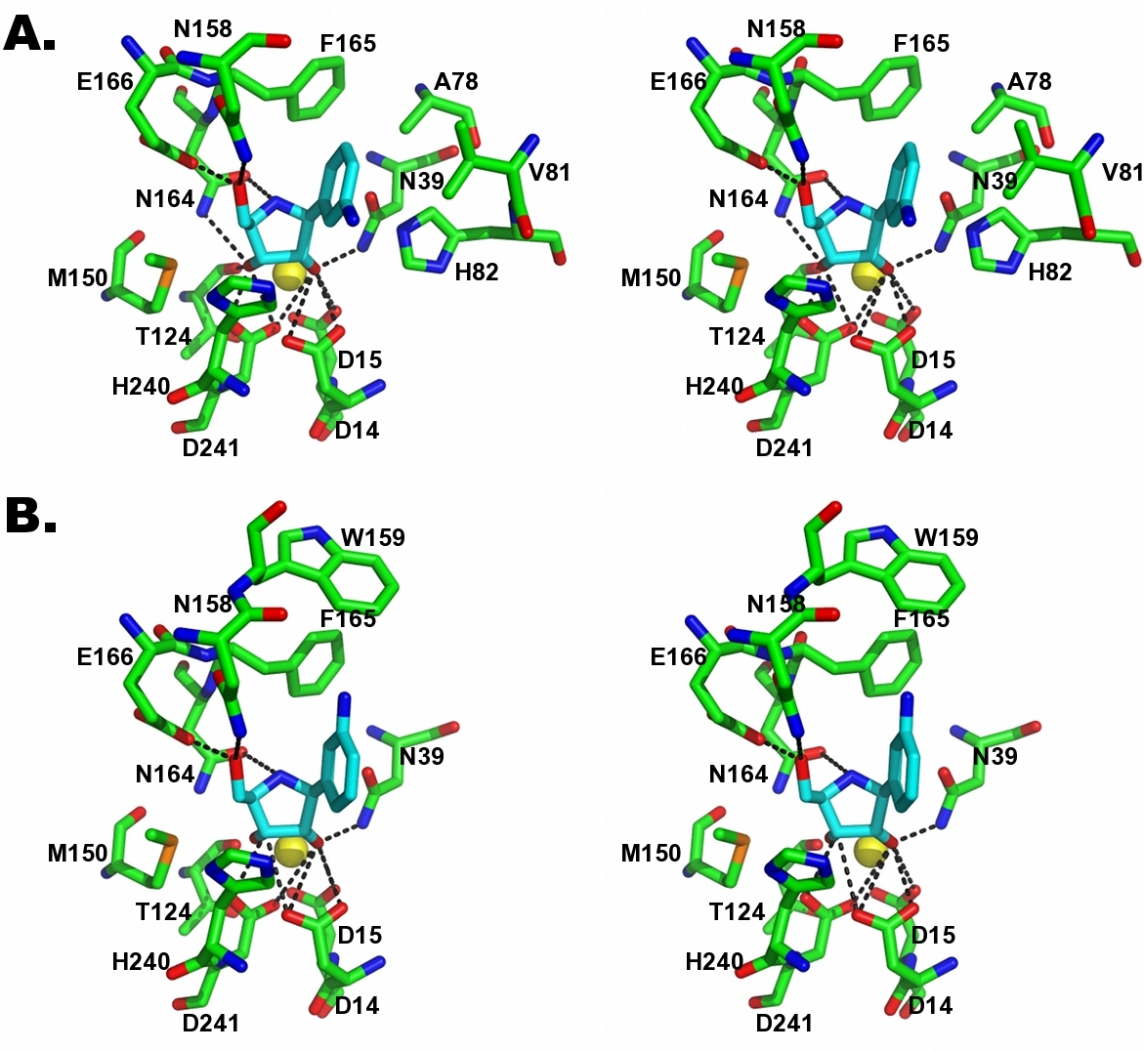
**Dual mode of DAPIR binding to RihA**. A) Stereo view of the active site of molecule A, showing the closed αL segment with the inhibitor N3 group pointing towards helix α9. B) Stereo view of the active site of molecule B, where the αL segment is flexible and the N3 amino group of DAPIR points in the opposite direction compared to panel C.

The diaminophenyl ring of the inhibitor, mimicking the nitrogenous base of substrates, establishes only van der Waals' interactions with the side chains of Ala78, Val81, Phe165, Phe223, and Lys228. No specific polar or hydrogen bonding interactions are formed between the amino groups of DAPIR and RihA, regardless of the inhibitor conformation. The further absence of the O2-carbonyl that is present in the natural pyrimidine nucleoside substrates may explain the dual binding mode of DAPIR at the active site. The interaction with DAPIR induces different degrees of rearrangement of the catalytic pocket of RihA. Indeed, in chains B and D, both the C-terminal portion of the α9 helix and the αL loop remain flexible as seen in the RihA-product complex (Fig. 3A). Conversely, the inhibitor conformation observed in molecule A of the RihA tetramer has a stabilizing effect on the protein conformation. In this polypeptide chain, residues Asn80-Val81-His82-Gly83 assume a helical conformation (αL), allowing the side chains of residues Val81 and His82 to face the inner portion of the active site and interact with the aromatic ring of DAPIR. Simultaneously, Val81 approaches also the C-terminal portion of the α9 helix, promoting van der Waals' interactions with amino acids Phe223 and His227. The closed conformation of the αL and α9 segments is apparently attainable only when the N3 amino group of DAPIR points towards the α9 helix. In this conformation, residue Ala78 closely approaches the C5 atom of the aminophenyl ring (3.8 Å). In the opposite orientation, the N3 amino group prevents Ala78 to reach the same conformation, and this likely destabilizes the αL segment (Fig. 4A and 4B). In the closed structure, the highly conserved His82 residue of the αL loop is interacting via a hydrogen bond between its Nδ side chain atom and the corresponding atom of His13, in a side-by-side orientation. These interactions, together with the intersubunit interactions described previously, allow a partial restructuring of helix α9 in a rotated and extended conformation. However, the terminal portion of the α9 helix and subsequent loop are only defined in molecule C, and this is likely due to crystal contacts established by this subunit. Indeed, the RihA-ligand interactions are apparently insufficient to induce the complete closure of the active site above the bound ligand and the shielding of the reaction centre from the bulk solvent, as previously observed in the *C. fasciculata *IU-NH bound to the structurally-related compound pAPIR [24].

The conformation of the iminoribitol moiety is characterized by a C4'-endo puckering of the ring, similar to that observed in other NH-ligand complexes [16,24,28]. This conformation is observed at low frequency in nucleosides and nucleotides in solution, where the C2'-endo or C3'-endo structures are more common, and is a direct consequence of the specific interactions between the ribosyl hydroxyls and the active site Ca^2+ ^ion. The orientation of the inhibitor moiety mimicking the pyrimidine base is roughly perpendicular to the iminoribitol ring, in an axial conformation that is also disfavoured in solution (Fig. 4A and 4B). Taken together, it is clear that the enzyme-substrate contacts at the CU-NH active site cooperate in stabilizing a high energy conformation of the ligand. This finding confirms that the enzymatic strategy by CU-NHs takes advantage of ground state destabilization to reach the transition state geometry, as demonstrated for the homologous IU-NHs.

Residues involved in the formation of helix αL are highly conserved in both IU- and CU-NH sequences, suggesting that this secondary structure element is likely a conserved feature in all CU-NH-substrate complexes. Conversely, the number and nature of the residues at the C-terminal portion of the α9 helix are highly variable among group I members, and can thus assume helical structures differing in length. Interestingly, strand β8 that immediately follows the α9 helix and pairs with β7 is present in CU-NHs so far characterized, but not in the non-specific IU-NHs. The presence of strand β8 is associated with the insertion of a glycine residue in position 236 (according to the RihA sequence numbering) and a proline in position 238, and can thus have effects on the mobility of helix α9.

### Emerging principles of nitrogenous base specificity in CU-NHs

**Figure 5:**
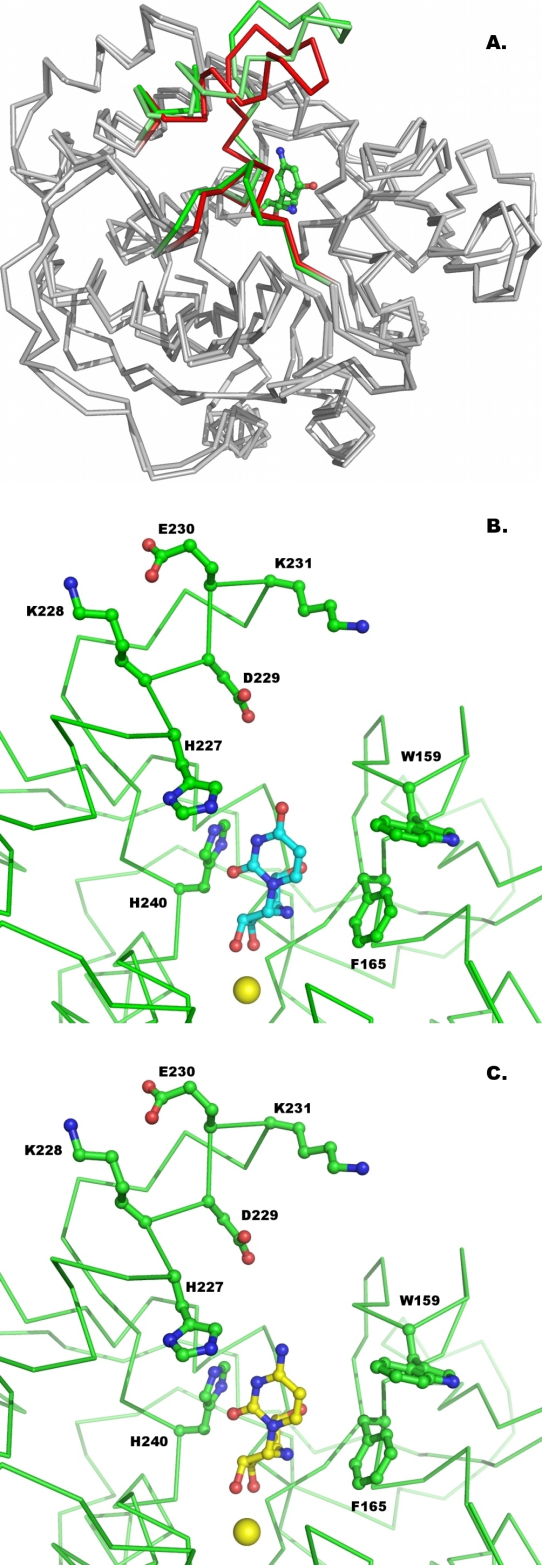
**A comparison of the structures of the pyrimidine-preferring CU-NHs RihA and RihB from *E. coli***. A) Superposition of the RihA and RihB monomers, shown as Cα traces. The α9 helix conformation differs significantly between the two proteins and is indicative of the plasticity of the flexible regions in NHs to adapt to different ligands. B) Model of interactions between RihA and uridine (top) or cytidine (bottom).

RihA and RihB are two CU-NH enzymes from *E. coli *displaying similar kinetic parameters for the steady state hydrolysis of the pyrimidine nucleosides uridine and cytidine. The structural comparison between the DAPIR-bound RihA and the available RihB structures helps the identification of the determinants for pyrimidine base recognition. A superposition of the closed form of *E. coli *RihA and RihB enzyme (rmsd = 1.4 Å for 304 Cα atoms of subunit A) highlights both conserved features and marked diversities at the active site. The residues of RihA involved in the αL formation are similarly arranged in RihB, as expected from their low variability in group I NHs. The conservative substitution of a valine with an isoleucine residue at position 81 in RihB maintains the characteristics of the van der Waals' interaction with the hydrophobic base of nucleosides, and does not affect significantly the conformational restraints imposed on the substrate. Thus, it is clear that the αL helix in group I NHs performs a conserved role. In contrast, two major differences are apparent when comparing the α9 helices of the two enzymes. The presence or absence of two specific Tyr residues in this element determines the purine versus pyrimidine nucleoside specificity in group I NHs [16]. The helix α9 superimposes well in the structures of RihA and RihB for the initial 15 residues (Gly210-Leu224 of RihA), but assumes a distinct orientation and extension of the protein backbone at its C-terminal region (Fig. 5A). In this region, the two enzymes have different number and type of residues, and do not show a clear homology or conservation. Secondly, the only polar residues positioned in the active site that can contribute to specific hydrogen bonding interactions for ligand discrimination are Lys228 in RihA, and Gln227 and Tyr231 in RihB. All these amino acids reach inside the active site only when helix α9 assumes the extended and ligand-stabilized closed conformation. Thus, the two enterobacterial CU-NHs clearly differ at important active site residues, yet these differences do not compromise their ability to interact and hydrolyze pyrimidine ribonucleosides.

The structure of the complex RihA-DAPIR reported here provides now a valid template for understanding the interactions of uridine and cytidine in both RihA and RihB, and helps clarifying the different catalytic efficiencies of the two enzymes for different pyrimidine substrates. When modelling the complex of RihA with uridine and cytidine, the charged His227 residue closely approaches the substituent present at the 4 position of the nitrogenous base (Fig. 5B). An acceptor group at this position, such as the 4-carbonyl of uridine, can establish favourable interactions with the charged lysine residue. Conversely, the enamino group of cytidine primarily acts as a hydrogen bond donor, and thus is disfavoured in the interaction with His227 (Fig. 5B). Indeed, RihA shows higher affinity for uridine than for cytidine (K_M _= 83 μM and 524 μM, respectively), and this can be accounted for by the different energetics of the interaction of the substrates with His227. Moreover, it should be noted that Lys228 may further rearrange in order to bring its side chain into the active site upon binding of natural substrates. This effect can also be appreciated when comparing the pseudo second order rate constant k_cat_/K_M_, indicative of the level of transition state stabilization in enzymatic reactions. Based on this parameter, RihA attains a greater degree of stabilization for uridine than cytidine (k_cat_/K_M _= 2 × 10^5 ^and 2 × 10^4 ^M^-1^s^-1^, respectively) [19], hence suggesting that the interaction with His227 may also contribute to the stabilization of the negative charge developing at the uracil ring along the reaction coordinate. Residue His240 is poised for proton transfer to the leaving group, or to further polarize the nitrogenous base via a hydrogen bond. In the RihB CU-NH, the residue pair Gln227/Tyr231 is positioned in the active site, and can establish polar interactions with the nitrogenous base that can guide substrate discrimination. RihB is similarly active with both uridine and cytidine (k_cat_/K_M _≈ 2 × 10^5 ^M^-1^s^-1^) [11], with a higher affinity for uridine over cytidine (K_M _= 142 μM and 532 μM, respectively) indicating again suboptimal binding interactions between the cytidine enaminic group and residues Gln227/Tyr231. However, while the RihA turnover number is similar for these two pyrimidinic substrates (k_cat _≈ 12 and 14 s^-1^), RihB hydrolyzes cytidine at a twofold faster rate (k_cat _≈ 12 vs. 5 s^-1^), indicating that in the presence of cytidine the Gln227/Tyr231 pair allows a higher degree of stabilization of the transition state, possibly by favouring the orientation of the O2 carbonyl for proton transfer. Taken together, these structural and kinetic data strongly support the hypothesis that CU-NHs do not discriminate different pyrimidine ribonucleosides during the substrate binding event, but mainly through the stabilization of the transition state of the enzymatic reaction. This finding parallels the recent results demonstrating that CU-NHs bind to purine nucleosides, but lack the crucial residues for efficient leaving group activation [16]. The similarities between the so far characterized CU-NHs RihA and RihB also suggest that the catalytic power of the enzymes is mediated by the conserved αL loop and the proton donor His239, while helix α9 provides a fine tuning of the substrate specificity through hydrogen bonding interactions.

### Active site plasticity

**Figure 6:**
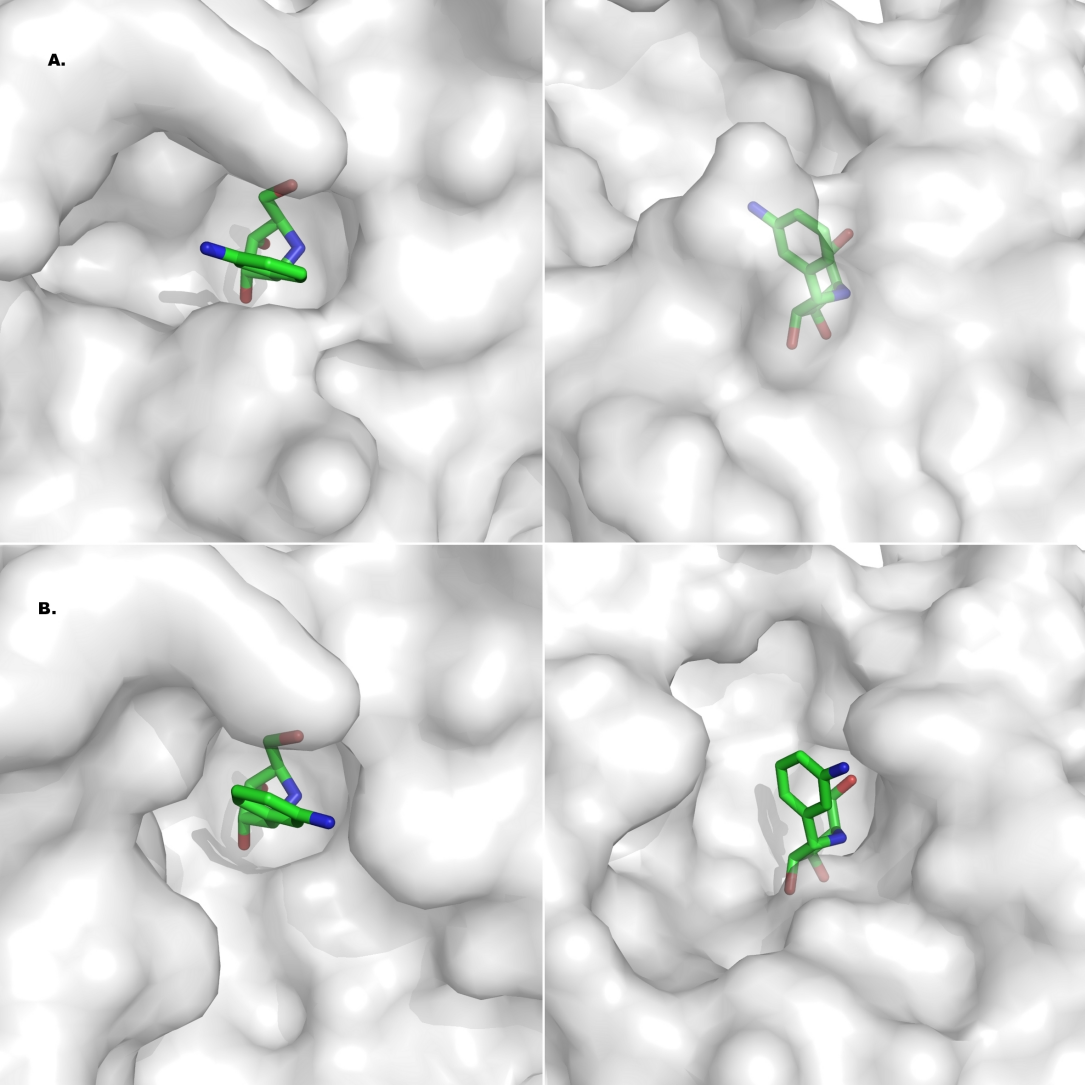
**Active site versatility of RihA**. Molecular surfaces at the RihA active sites of two independent molecules. Two orthogonal views are shown, highlighting the existence of empty cavities that may accommodate different types of substrates. Panels A) and B) depict the surfaces of chain A and chain D, respectively.

We further analyzed the binding cavity of RihA to assess whether it is tailored for the common RNA nucleosides, or may display a broader substrate specificity. The molecular surface of the active site does not show a tight complementarity to the DAPIR molecule, in either conformation (Fig. 6A and 6B). Indeed, the enzyme does not fully wrap around the ligand, rather leaving empty spaces on both sides of the ring. These unfilled cavities may allow other nucleosides to act as substrates. Indeed, substituents protruding from the C2, N3, and C4 positions could be accommodated in the binding site without major structural rearrangements. Substituents at C5 are likely restricted to methyl or similarly small groups, since larger moieties would suffer from the steric hindrance of residues Ala78 and Trp159, preventing the rearrangement of the αL loop to the catalytically-relevant conformation as observed in the present structure in chains B, C, and D. The molecular surface of the enzyme clearly shows that the binding cavity maintains open spaces, and thus may accommodate substrates bearing a bulkier aglycone compared to pyrimidine bases such as uracil or cytidine.

### Catalytic cycles between the open and closed conformations of RihA

The availability of highly detailed structures of DAPIR and ribose bound to the RihA CU-NH provide insights into the dynamic enzyme rearrangements accompanying N-glycosidic bond hydrolysis. One open question in NH enzymology is how is the transition from the closed to the open form of RihA triggered upon catalysis? Cleavage of the N-glycosidic bond of pyrimidine nucleosides allows the relaxation of the constrained nucleoside structure observed in the RihA/DAPIR complex, and allows a relocation of both the base and the ribose products. A repositioning of the nitrogenous base, required to avoid repulsive interactions with the ribose C1' atom, may disrupt its contacts with the active site residues from both αL and α9. As the base moves away from the side chain atoms of Val81 and His82, the αL conformation will become less stable, or could sample different conformations as demonstrated by the unliganded or product-bound group I NH structures. This flexibility may lead to the disruption of the hydrogen bond between His13 and His82, allowing the side chain of the former amino acid to protrude in the active site and preventing residue Phe223 to assume the conformation observed in the RihA-DAPIR complex. When the αL is no more present in the active site, also the van der Waals' contacts between Val81 and Asp88 from αL, and Phe223 and His227 in helix α9 cannot be established (Fig. 4A and 4B). Hence, the α9 helix is destabilized in absence of the closed αL segment and undergoes a rotation about its axis and a swivelling movement towards the open conformation. The lack of specific stabilizing contacts results in a high flexibility of this segment, typically disordered in group I NH structures determined by X-ray crystallography. The side chain of Asn39 undergoes a minor rotation allowing the ribose ring to move by about 0.5 Å towards the outer portion of the active site, in a weakly bound conformation that can be displaced by the bulk solvent. Thus, the conformational rearrangements occurring to αL and α9 upon release of the nitrogenous base appear to be coupled, since in the close conformation the two structural elements display strong reciprocal interactions, as well as with the substrates. Further experiments to confirm this cooperative motion are underway using mutagenesis and molecular dynamics calculations. Given the importance of these motions in regulating product release, the present findings also raise the question as to whether the steady-state kinetic parameters are affected by the isomerization step, as seen for the group II IAG-NH from *Trypanosoma vivax *[29].

## Discussion

The work here reported provides the first description at the atomic level of the interactions of a pyrimidine-specific NH enzyme with a competitive inhibitor that resembles the natural substrates. The comparison between this structure and the same enzyme bound to the reaction product helped the definition of several aspects of the enzymatic mechanism and dynamics in CU-NHs. Moreover, it provides insights into future developments for the identification of NH substrates alternative to classical RNA nucleosides.

The catalytic mechanism of group I NH enzymes, as determined for the *C. fasciculata *IU-NH, proceeds through an S_N_1-type transition state with an oxonium ion-like geometry at the ribose ring [2,27]. In RihA, substrate binding induces the active site closure, and the correct positioning of active site key residues for substrate recognition. The calcium metal centre directs the binding of the substrate molecule and its distortion to a high-energy conformation, and may activate the coordinating water molecule for nucleophilic attack. The involvement of the Asp10 as general base in the mechanism has been well documented [2,24]. After hydrolysis, the enzyme active site opening leads to the base release and an accessible active site. Comparing the substrate-bound and ribose-bound RihA structures, three ordered water molecules displace the nucleobase in the latter, thus supporting the hypothesis that the release of ribose in the final step of the catalytic cycle requires its displacement by bulk solvent molecules. The availability of the structures of the product-bound and ligand-bound RihA enzyme now allows the use of molecular dynamics simulations to provide further insights into the conformational rearrangements taking place in the catalytic cycle of CU-NHs.

The positions of amino acids His82, Lys228, and His240 are highly indicative of their active role in the stabilization of the Michaelis complex. His240 is potentially involved in the transfer of a proton to the pyrimidine carbonyl O2, since mutation of the homologous histidine in RihB suggested indeed an essential role in leaving group activation. Residues His227 and Lys228 likely assists in the binding of nucleosides, establishing hydrogen bonds with the nucleobase substituents that direct substrate discrimination and may also participate in catalysis. In light of the present structure, we can argue that the main role of His82 is to act as (sensor) of the presence of a substrate in the active site, and to initiate αL and α9 restructuring after establishing a side-by-side H-bond connection with His13. This interaction allows the relocation of the α9 helix residues by locking His13 in an orientation that removes the side chain from the active site. The catalytic activity of group I NHs shows a bell-shaped pH dependency, involving two residues with pKa nearing neutral for maximal activity. Based on the present results we speculate that protonation state of His82 may affect the kinetics of transition between the open and closed structures.

RihA and RihB differ in the conformation of the α9 helix and at the amino acids believed to mediate substrate specificity, yet their steady state kinetic parameters are fairly similar. The DAPIR-RihA complex allowed us to explain this apparent discrepancy disclosing that the enterobacterial enzymes perform hydrolysis of ribonucleosides through a transition state that can be stabilized via substantially different interactions within the active site. Structural models obtained using natural occurring substrates support this hypothesis and suggest that substrate discrimination occurs after the initial substrate binding event, when the enzyme restructures to maximize binding interactions for catalysis. Instead, the chemical step is carried out with similar efficiency by the conserved residues at the active site. In turn, this implies that the steady state rate constant for pyrimidine nucleoside hydrolysis may be significantly affected by the kinetics of the open-closed transition in the enzyme. The functional implication of these findings is that the two CU-NH enzymes from *E. coli *can both guarantee efficacy of catalysis for different substrates. Moreover, the presence of two distinct enzymes with different ability of interaction and active site flexibility might assure a wide versatility in substrate recognition.

The preference of RihA for uridine is consistent with the fact that in the pyrimidine salvage pathway of *E. coli *cytidine is efficiently converted to uridine by cytidine deaminases, a family of enzymes that has been discovered with the ability to deaminate cytidines also directly on RNA[30,31]. However, the recycling of uridine and cytidine in *E. coli *is normally promoted by reversible nucleoside phosphorylases, hence the activity of RihA and RihB is likely directed also towards alternative N-ribosides. They might act on common nucleosides under yet unidentified physiological conditions, or alternatively preferentially act on modified nucleosides found in RNA molecules. The present structure demonstrates that RihA is endowed with a significant active site flexibility, as demonstrated by the dual binding mode of DAPIR and the different conformations attainable by the enzyme, and thus may also act on modified nucleosides. Among these, pseudouridine (ψ) contains a C-glycosidic bond, and is thus not a potential substrate of CU-NHs. Um and Cm are rare nucleotides with methyl groups at the O2' position, and are also likely not substrates for NHs since this modification would disrupt the crucial interaction with the active site Ca^2+ ^ion that is necessary for substrate binding and distortion. On the contrary, molecular modelling analysis suggests that the common bacterial base-modified methyl-containing (m^3^C, m^5^C, m^5^U), sulphur-containing (s^2^U, s^4^U), and also rare Se^2^U and D nucleosides, are all able to fit the active site of RihA and RihB, and thus raising the possibility that potential substrates of CU-NHs could likely belong to this group of RNA modifications, which are essential for normal cell growth. Interesting, methylations of RNA affect in particular unusual base-pairing interactions of ribosomes, and their accumulation in bacteria is often indicative of emerging antibiotic resistance [32]. Thiopyrimidine analogues and seleniumuridine are present at specific positions in specific tRNA molecules, and are involved in unusual activities such as a bacterial photosensor or thermosensor [13]. In addition, the processes following thiouridine excitation by UV-A light are related to deleterious effects induced in living systems. Thus, resistance to exogenous compounds, ambient sensitivity, and detoxification from harmful pyrimidines, appear conditions where the hydrolytic function of bacterial CU-NHs might operate. We expect that further studies on the activity of these enzymes may eventually confirm their involvement in the turnover of modified nucleobases deriving from the breakdown of RNA molecules.

## Conclusions

The structure of RihA with the inhibitor DAPIR describes the enzyme structural rearrangement and interactions when bound to an active site ligand, underscores a buttressing role of the tetrameric assembly, and provides a snapshot of a structure sampled along the reaction coordinate. A comparison with the ribose-bound form reveals that the presence of the nitrogenous base is required for the enzyme active site closure. The flexible loops in the αL and α9 regions of group I NHs can assume different conformation to adapt to the active site ligand properties. The conserved His82 residue of group I NHs aids the restructuring of the active site through interaction both with the substrate and protein residues. The similar catalytic efficiencies of the two enterobacterial CU-NHs may reflect a rate-limiting role in catalysis of the observed conformational changes.

## Methods

### Protein cloning, expression and purification

The *rihA *gene (936 bp) was amplified from *E. coli *genomic DNA extracted from the K12 derivative DH5α strain. The gene was amplified by PCR using the forward primer *rihA*-NdeI (TACGTGCATATGGCACTGCCAATT) and reverse *rihA*-BglI (GGGCAGATCTTTAAGCGTAAAATTTCAG), designed to add the NdeI restriction site at the 5' end of the gene, and the BglI site after the stop codon. Amplification was performed with the Deep Vent DNA Polymerase (New England Biolabs) in the presence of 0.2% DMSO to improve DNA denaturation. The gene was cloned via blunt end ligation in the pBluescript II SK (-) vector (Stratagene) previously treated with SmaI, and sequenced on both DNA strands using the automated dideoxy method. The pBSK-*rihA *plasmid was treated with the restriction endonucleases NdeI and BglI, the fragment containing the *rihA *gene was purified from 1% agarose gel, and ligated between the corresponding sites of the pET-28b(+) vector (Novagen). A liquid LB overnight culture of BL21(DE3) *E. coli *cells transformed with the pET28-*rihA *was diluted 1:100 in LB medium containing 1% glucose and 50 μg/ml kanamycine. Expression was induced at a culture optical density at 600 nm of 0.6 by addition of 0.5 mM IPTG for 3 h at 37°C under vigorous shaking. Cells were harvested by centrifugation, resuspended in a buffer containing 20 mM Tris pH 8, 50 mM NaCl, and the Complete EDTA-free mixture of protease inhibitors (Roche), and disrupted by sonication. The RihA protein in the soluble fraction was bound to a NiNTA resin (Qiagen) and eluted using a buffer containing 100 mM Tris pH 8, 500 mM NaCl and 500 mM imidazole. The enzyme was further purified using size exclusion chromatography on a Superdex 200 column using an isocratic elution with 50 mM Hepes pH 7.2, and 150 mM NaCl. The N-terminal hexahistidine peptide was removed by partial proteolysis using thrombin at a ratio of 1:500 w/w, and the protein further purified using size exclusion chromatography.

### Inhibition studies

The inhibition constant of DAPIR was measured comparing the initial rate of the RihA-catalyzed hydrolysis of uridine in the presence or absence of inhibitor. Reaction were carried out in Hepes buffer pH 7.4, 100 mM NaCl and 80 μM uridine. Progress curves were recorded on a UltroSpec2100 spectrophotometer (GE Healthcare) at 37°C with a wavelength of 262 nm (Δε = -2.18 μM^-1^·s^-1^). Initial velocities were measured in the presence of different concentrations of inhibitor, and the inhibition constant was derived from the equation v0/vI = (K_M_(1 + [I]/K_I_) + [S])/(K_M _+ [S]), where v0 and vI are the initial rates in the absence and presence of inhibitor, respectively. This equation assumes competitive inhibition, and this is supported by the structural resemblance of DAPIR to the natural substrates and the observed binding at the active site residues.

### Crystallization and data collection

A 8 mg·ml^-1 ^RihA solution in 50 mM Hepes pH 7.2, 150 mM NaCl was mixed with a 5:1 molar excess of DAPIR solubilized in 50 mM Hepes pH 7.2, and incubated at 4°C for 3 hours. Crystals of the RihA-DAPIR complex were obtained by hanging drop vapour diffusion at 20°C. The protein/inhibitor complex was mixed with equal volumes of a precipitant solution containing 25% PEG 4000, 0.1 M sodium acetate pH 5.0. Crystals were visible within three days, and grew to maximum dimensions of 300 × 150 × 150 μm. For data collection, a single crystal was mounted in a nylon-fiber loop and flash-cooled in a dry nitrogen stream at 100 K. A buffer with the PEG concentration increased to 34% was used as cryoprotectant. Diffraction data to 2.05 Å were collected at the beamline ID14-EH1 of the European Synchrotron Radiation Facility (Grenoble, France) on a Quantum4 CCD detector using the oscillation method and an X-ray wavelength of 0.933 Å. Data were indexed, integrated, and scaled using the program XDS [33], and the measured intensities were converted to structure factor amplitudes using the program TRUNCATE [34], part of the CCP4 suite [35]. The crystals belong to the monoclinic P2_1 _space group, as judged from systematic absences, with unit cell dimensions a = 84.36 Å, b = 82.99 Å, c = 94.00 Å, β = 112.1°. The cell volume is consistent with four RihA monomers in the asymmetric unit, with 44% solvent and a Matthews' coefficient of 2.3 Å^3^/Da.

### Structure solution and refinement

The structure of RihA was solved using the molecular replacement technique as implemented in the program MOLREP [36] using the monomer of the RihA from the YbeK/ribose complex structure (PDB code 1YOE) as the search model after removal of all solvent molecules, ligands, and ions. A single conformation was chosen for the residues that displayed multiple rotamers in the RihA-ribose complex structure. Rotation and translation functions calculated using data to 4.0 Å resolution yielded four unambiguous, distinct solutions in space group P2_1 _but not in space group P2, confirming the initial assignment. An initial electron density map was calculated with |2F_o_-F_c_| coefficients and model phases using data to 2.1 Å resolution. This map showed the presence of the catalytic Ca^2+ ^ions, and indicated that one DAPIR molecule was bound at each active site. Manual rebuilding in sigmaA-weighted (2mF_o_-DF_c_, ϕ_c_) and (mF_o_-DF_c_, ϕ_c_) electron density maps using the program COOT [37] and restrained maximum-likelihood positional and isotropic temperature factor refinement with REFMAC5 [38] were performed in a cyclic process to gradually improve the model. After the R-factor decreased below 0.30, water molecules were added to the model by visual inspection in positive peaks >3σ of difference maps. Anisotropic motions were modelled through refinement of the torsion, libration, and screw axis tensors (TLS), treating each independent polypeptide in the asymmetric unit as an independent group. The final crystallographic R_crys _and R_free _values converged to 0.20 and 0.24, respectively. The geometry of the model was monitored during refinement with PROCHECK [39] and MOLPROBITY[40]. Molecular superpositions were carried out with the program LSQMAN[41]. Figures and schemes were generated using PYMOL http://www.pymol.org and IsisDraw. The coordinates and structure factors of the RihA-DAPIR complex have been deposited with the PDB http://www.rcsb.org/pdb with accession code 3G5I.

## Authors' contributions

GG collected the diffraction data, participated in the structural analysis, and drafted the manuscript; LM planned experiments and cloned the RihA gene; PT expressed, purified, and crystallized the complex; and MD designed experiments, solved and refined the structure, performed part of the structural analysis, and wrote the manuscript. All authors read and approved the final version of the manuscript.
